# Supplementary material for: Amino acid biostimulant increases radiata pine photosynthetic efficiency and growth with shifts in mycobiome and nitrogen assimilation
Source: Environ Microbiome. 2025 Dec 14;21:15. doi: 10.1186/s40793-025-00835-x (PMC12821856; doi:10.1186/s40793-025-00835-x)
Supplement: Supplementary file 2 — Supplementary Material 2 [file 40793_2025_835_MOESM2_ESM.docx]

**Figure S1**


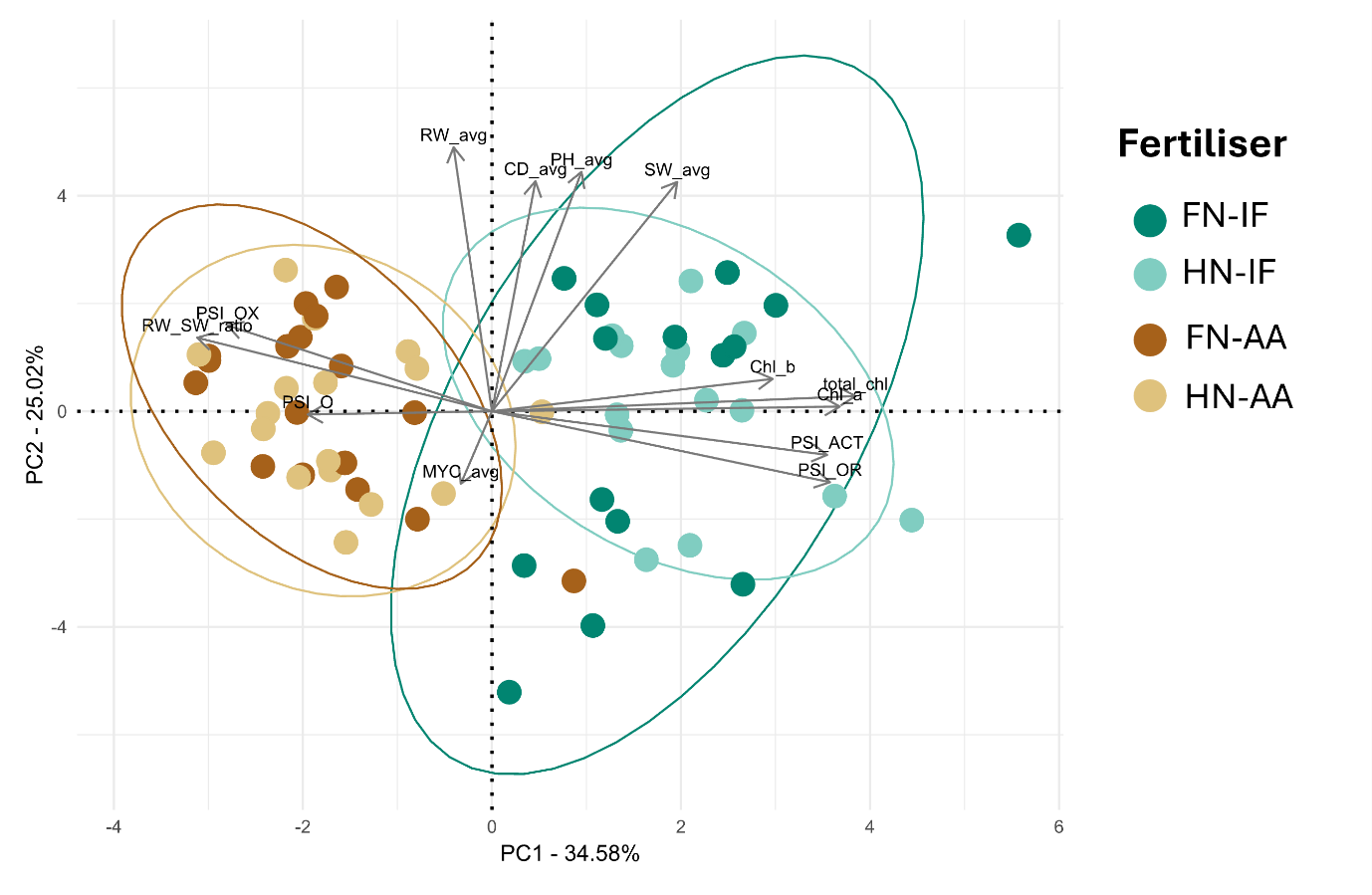


**Figure S1:** Principal Component Analysis (PCA) biplot of morphological and photosynthetic variables measured under four different fertiliser treatments. Dots represent individual samples, colored and enclosed by ellipses indicating the 95% confidence region for each fertiliser group. The PC1 and the PC2together explain 34.58 % and 25.02 % of the total variance, respectively. The vector's direction and length indicate the traits' contribution to the first two components in the PCA. Morphological variables include shoot weight (SW_avg), plant height (PH_avg), collar diameter (CD_avg), and root–shoot ratio (Rw_SW_ratio), while photosynthetic traits include total chlorophyll (total_chl), chlorophyll a (Chl_a), chlorophyll b (Chl_b), and PSI activity measurements.

**Figure S2**


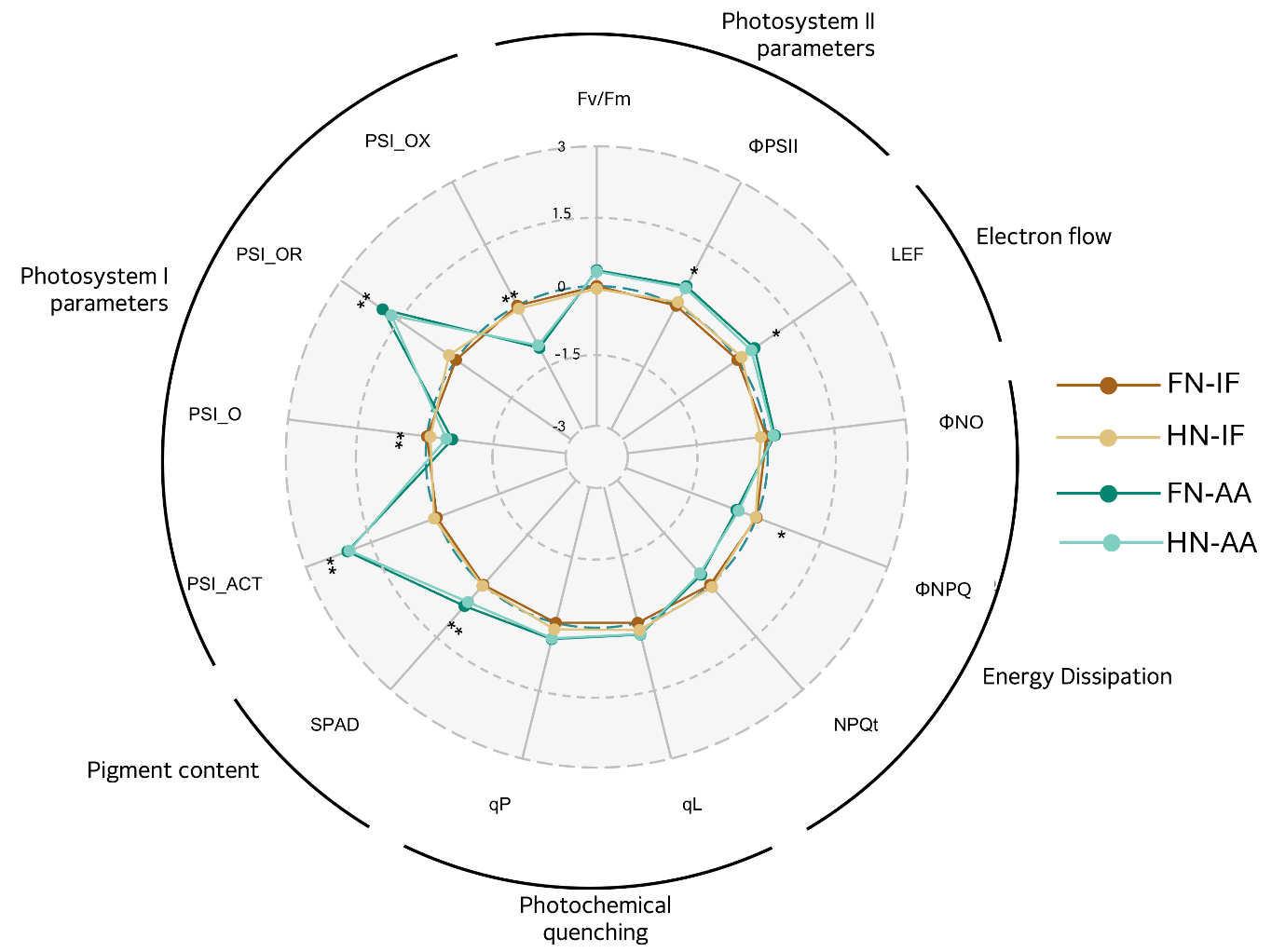


**Figure S2:** Effect of amino acid fertigation on photosynthetic activity.

A radar plot shows the effects of fertilizers on selected chlorophyl fluorescence parameters. The value of each parameter represents the effect size (Glass’s Δ) calculated against control treatement (FN-IF). Metrics include Fv/Fm and light-acclimated parameters (ΦPSII (Y(II)), LEF, ΦNO, ΦNPQ, NPQt, qL, qP), pigment/greenness (SPAD), and PSI indices (PSI_O, PSI_OR, PSI_OX, PSI_ACT). These metrics are presented strictly as descriptive performance indicators; detailed interpretation is not attempted here.** indicates p-value <0.01, * indicates p-value < 0.05.

**Figure S3**


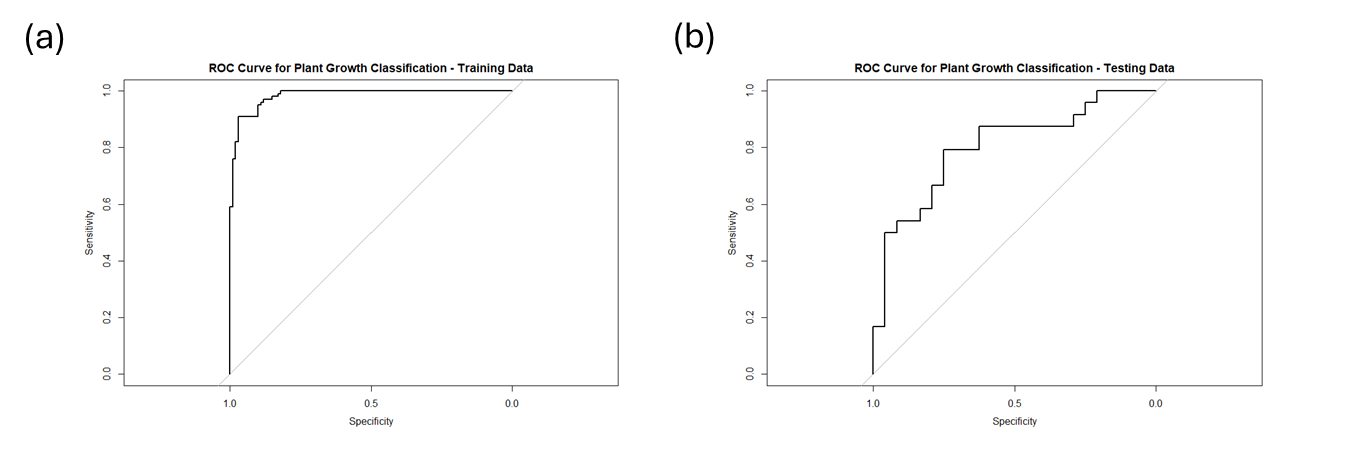


**Figure S3:** Gredient boosting model performance. ROC curve for the classification of plant growth conditions based on the training dataset, showing high predictive performance (AUC = 0.983). (c) ROC curve for the classification of plant growth conditions on the testing dataset, demonstrating a reasonable predictive accuracy (AUC = 0.802).

**Figure S4**


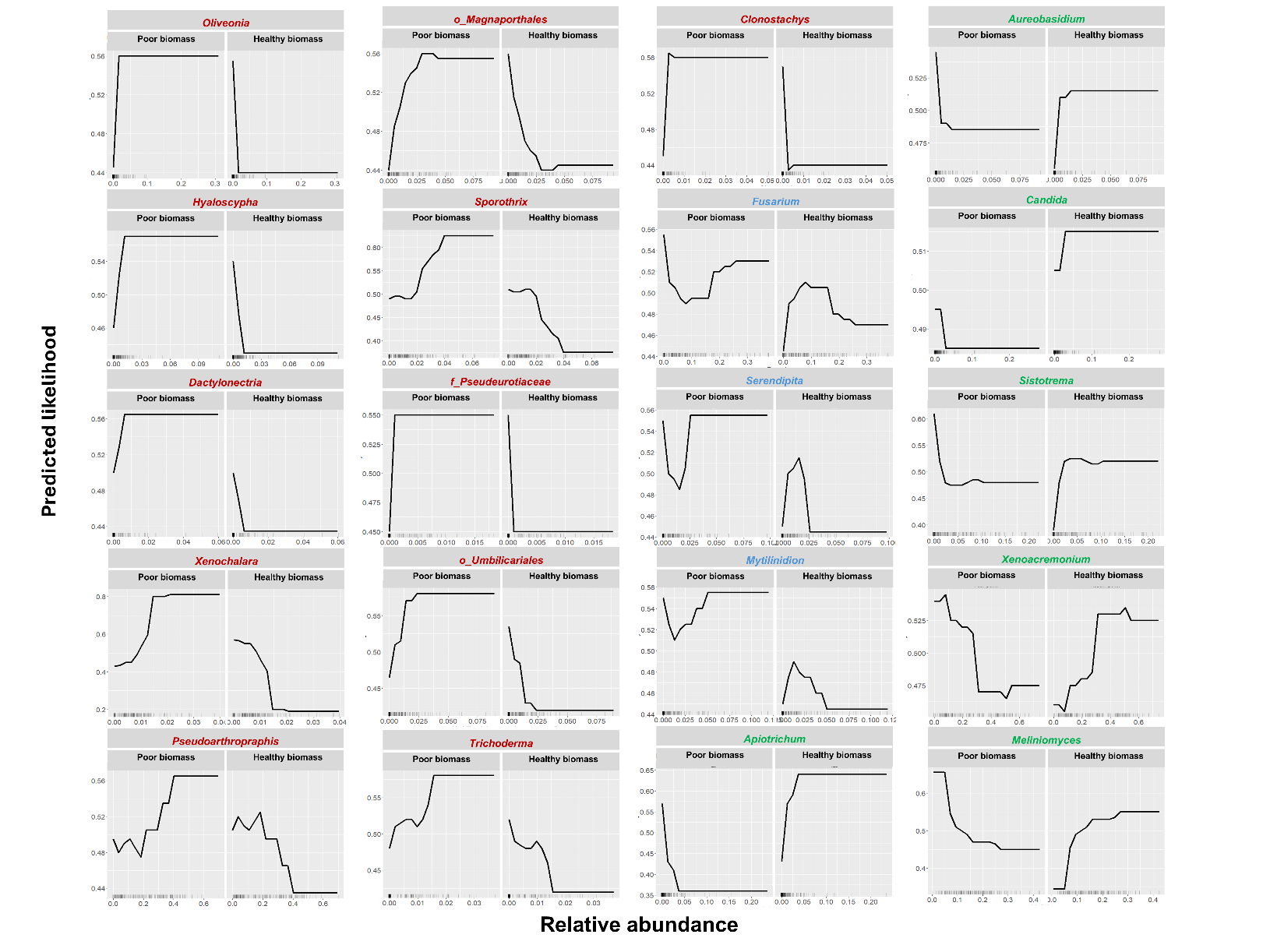


**Figure S4:** Full partial-dependence gallery for all remaining taxa included in the gradient-boosting classifier. Panels use the same axis scales, smoothing settings and uncertainty display as Fig. 6B. Taxon-label colour encodes predominant association (green = positive; red = negative; blue = non-monotonic/mixed). Prefixes “o_” and “f_” indicate order- and family-level assignments where genus was not resolved. PDPs illustrate associations within this dataset and are not evidence of causality.
